# The Prognostic Impact of Age at Diagnosis Upon Breast Cancer of Different Immunohistochemical Subtypes: A Surveillance, Epidemiology, and End Results (SEER) Population-Based Analysis

**DOI:** 10.3389/fonc.2020.01729

**Published:** 2020-09-23

**Authors:** Shibin Cai, Wenjia Zuo, Xunxi Lu, Zongchao Gou, Yi Zhou, Pengpeng Liu, Yin Pan, Shuzheng Chen

**Affiliations:** ^1^Department of Breast Surgery, Lishui Hospital, Zhejiang University School of Medicine, Lishui, China; ^2^Department of Breast Surgery, Fudan University Shanghai Cancer Center, Shanghai, China; ^3^Department of Oncology, Shanghai Medical College, Fudan University, Shanghai, China

**Keywords:** breast cancer, mortality, immunohistochemical subtype, age at diagnosis, prognosis

## Abstract

**Background and Objectives:** The influence of age at diagnosis of breast cancer upon the prognosis of patients with different immunohistochemical (IHC)-defined subtypes is still incompletely defined. Our study aimed at examining the association of age at diagnosis and risk of breast cancer-specific mortality (BCSM).

**Methods:** 172,179 eligible breast cancer patients were obtained for our study cohort using the Surveillance, Epidemiology, and End Results database from 2010 to 2015. Patients were classified into four IHC-defined subtypes according to their ER, PgR, and HER2 status. Kaplan–Meier plots were used to describe BCSM among patients in different age groups. A Cox proportional hazards model was used for multivariate analysis. A multivariable fractional polynomial model within the Cox proportional hazards model was used to evaluate the relationship between age at diagnosis and the risk of BCSM.

**Results:** For the whole cohort, the median follow-up time was 43 months. Patients younger than 40 years and those older than 79 years presented with the worst BCSM (hazard ratio [HR] 1.13, 95% confidence interval [CI] 1.03–1.23, and HR 3.52, 95% CI 3.23–3.83, respectively, *p* < 0.01, with age 40–49 years as the reference). The log hazard ratios of hormone receptor (HoR)(+)/HER2(–) patients formed a quadratic relationship between age at diagnosis and BCSM, but not in the other three subtypes of breast cancer. In the HoR(+)/HER2(–) subtype, patients younger than 40 years had worse BCSM than those aged at 40–49 years (HR 1.26, 95% CI 1.10–1.45, and *p* < 0.01).

**Conclusions:** Women diagnosed with HoR(+)/HER2(–) breast cancer younger than 40 years or older than 79 years of age suffer higher rates of cancer-specific mortality. Young age at diagnosis may be particularly prognostic in HoR(+)/HER2(–) breast cancer.

## Introduction

Breast cancer is the most common cancer in females. In 2020, it is estimated that 276,480 new breast cancer cases and 42,170 breast cancer deaths will occur in the United States alone (1). Breast cancer is a heterogeneous disease, which the 2013 St. Gallen Consensus classified into four main molecular subtypes: luminal A, luminal B, HER2-enriched, and basal-like breast cancer (2–4). Molecular subtypes play an important role in guiding the clinical treatment of breast cancer, and many studies have been conducted upon the differences between different tumor subtypes. For example, luminal B tumor is more likely to express genes associated with high tumor proliferation compared to luminal A tumors (5). The different molecular subtypes of breast cancer have diverse biological phenotypes and varying degrees of response toward systemic treatments (2, 3, 5), thus showing different patterns of relapse and long-term prognosis (6, 7). Though the molecular classification of breast cancer requires using Gene Expression Profiling (GEP) and DNA microarrays to identify distinct subtypes, the use of GEP in routine clinical diagnosis is neither economically feasible nor practical. Therefore, immunohistochemical staining of estrogen receptor (ER), progesterone receptor (PgR), and human epidermal growth factor receptor-2 (HER2) can be used as surrogate to roughly determine four main subtypes for clinical application: “luminal A” (ER and/or PgR positive and HER2 negative), “luminal B” (ER and/or PgR positive and HER2 positive), “HER2-overexpressed” (ER and PgR negative and HER2 positive), and “Triple-negative” (ER, PgR, and HER2 negative).

Age at diagnosis has been reported to be an independent prognostic factor for breast cancer in several studies (8–11). The influence of age upon the prognosis of patients with different tumor subtypes is still incompletely defined. Young age seems to be a significant prognostic factor in women with luminal subtype breast cancers (12–14). In addition, studies have also indicated that in triple-negative breast cancer, age group of <40 years is significantly associated with poor prognosis (15, 16). However, many of these previous studies were of limited sample size. Therefore, our study aimed at examining the relationship between age at diagnosis and the risk of breast cancer-specific mortality (BCSM) using the largest study population possible from the Surveillance, Epidemiology, and End Results (SEER) database.

## Materials and Methods

### Data Source and Study Population

Data were obtained from the SEER database (www.seer.cancer.gov), which incorporates 18 population-based cancer registries (November 2016 submission). We enrolled eligible patients based on the following inclusion criteria (Supplementary Figure 1): female sex, unilateral breast cancer, only one primary breast cancer, year of diagnosis from 2010 to 2015, diagnosis not obtained from a death certificate or autopsy, age at diagnosis ≥20 years old, American Joint Committee on Cancer stages I–III, pathologic confirmation of invasive ductal carcinoma, and the known ER, PgR, and HER2 statuses. Due to the fact that HER2 status was not registered in the SEER database until 2010, only patients with breast cancer diagnosed after 2010 were included.

The status of ER, PgR, and HER2 was used to classify patients into four immunohistochemical (IHC)-defined breast cancer subtypes: hormonal receptor (HoR)(+)/HER2(–) group (ER-positive and/or PgR-positive and HER2-negative), HoR(+)/HER2(+) group (ER-positive and/or PgR-positive and HER2-positive), HoR(–)/HER2(+) group (ER-negative, PgR-negative, and HER2-positive), and triple-negative group (ER-negative, PgR-negative, and HER2-negative).

For this study, the follow-up time was calculated from the time of first diagnosis for breast cancer. The primary study outcome was BCSM, and it was defined as the time from the initial breast cancer diagnosis to the death of the patient from breast cancer. Patients who died of other causes were censored upon their date of death.

### Statistical Analysis

The patient demographics and tumor characteristics of this study are provided in Table 1. Variables classified by IHC-defined breast cancer subtype were compared using the χ^2^ test. The reverse Kaplan–Meier method was used to calculate median follow-up time. Breast cancer-specific survival in the different age groups was described using Kaplan–Meier. Age at diagnosis was treated as a categorical variable classified into the following age groups: <40, 40–49, 50–59, 60–69, 70–79, and >79 years. The association of age group with the risk of BCSM was evaluated using the Cox proportional hazards model. Variables shown to be significantly associated with BCSM in the univariate analysis were included in the multivariate analysis. Adjusted hazard ratio (HR) with 95% confidence interval (CI) was calculated using the multivariable Cox proportional hazards model, while simultaneously controlling for clinical prognostic risk. To further determine whether there was a significant interaction between age at diagnosis and IHC-defined breast cancer subtype for predicting BCSM, we used an interaction term (i.e., age × subtype) and performed pairwise comparisons using different combinations of age and subtype. Age was treated as a continuous variable, and a multivariable fractional polynomial model within the Cox proportional hazards model was used to examine a potential nonlinear relationship between age at diagnosis and BCSM. The difference between the nonlinear and linear models was assessed using a likelihood ratio test to test for nonlinearity. A two-sided *p*-value of < 0.05 was considered to indicate statistical significance. All analyses were performed in STATA 15 (StataCorp, College Station, Texas, USA).

**Table 1:** Demographic and tumor characteristics of the patients.

**Characteristic**	**Total**	**HoR(+)/HER2(–)**	**HoR(+)/HER2(+)**	**HoR(–)/HER2(+)**	**Triple-negative**	***p-*value^**a**^**
	**(*n* = 172,179)**	**(*n* = 120,408)**	**(*n* = 20,643)**	**(*n* = 8,974)**	**(*n* = 22,154)**	
	***n* (%)**	***n* (%)**	***n* (%)**	***n* (%)**	***n* (%)**	
**Median follow-up:**	43 (26–62)	43 (26–62)	41 (24–61)	42 (24–61)	45 (27–63)	
**months (IQR)**						
**Age at diagnosis**						*p < * 0.001
<40	10,615 (6.2)	5,597 (4.7)	2,041 (9.9)	766 (8.5)	2,211 (10.0)	
40–49	31,831 (18.5)	20,901 (17.4)	4,594 (22.3)	1,754 (19.6)	4,582 (20.7)	
50–59	44,722 (26.0)	29,888 (24.8)	5,932 (28.7)	2,879 (32.1)	6,023 (27.2)	
60–69	45,487 (26.4)	33,574 (27.9)	4,679 (22.7)	2,087 (23.3)	5,147 (23.2)	
70–79	26,557 (15.4)	20,512 (17.0)	2,294 (11.1)	1,003 (11.2)	2,748 (12.4)	
>79	12,967 (7.5)	9,936 (8.2)	1,103 (5.3)	485 (5.4)	1,443 (6.5)	
**Race**						*p < * 0.001
White	134,203 (78.0)	96,409 (80.1)	15,657 (75.9)	6,407 (71.4)	15,730 (71.0)	
Black	19,326 (11.2)	11,038 (9.2)	2,441 (11.8)	1,270 (14.2)	4,577 (20.7)	
Other^b^	18,650 (10.8)	12,961 (10.7)	2,545 (12.3)	1,297 (14.4)	1,847 (8.3)	
**Marital status**						*p < * 0.001
Married	97,347 (56.6)	68,117 (56.6)	11,961 (57.9)	5,185 (57.8)	12,084 (54.6)	
Unmarried	66,529 (38.6)	46,482 (38.6)	7,742 (37.5)	3,368 (37.5)	8,937 (49.3)	
Unknown	8,303 (4.8)	5,809 (4.8)	940 (4.6)	421 (4.7)	1,133 (5.1)	
**Year of diagnosis**						*p < * 0.001
2010	25,387 (14.7)	17,592 (14.6)	2,931 (14.2)	1,354 (15.1)	3,510 (15.8)	
2011	27,250 (15.8)	19,209 (16.0)	2,991 (14.5)	1,352 (15.1)	3,698 (16.7)	
2012	28,336 (16.5)	19,866 (16.5)	3,342 (16.2)	1,448 (16.1)	3,680 (16.6)	
2013	29,434 (17.1)	20,745 (17.2)	3,564 (17.3)	1,467 (16.4)	3,658 (16.5)	
2014	30,205 (17.6)	21,097 (17.5)	3,768 (18.2)	1,602 (17.8)	3,738 (16.9)	
2015	31,567 (18.3)	21,899 (18.2)	4,047 (19.6)	1,751 (19.5)	3,870 (17.5)	
**Laterality**						*p < * 0.001
Left	87,132 (50.6)	60,537 (50.3)	10,532 (51.0)	4,664 (52.0)	11,399 (51.4)	
Right	85,047 (49.4)	59,871 (49.7)	10,111 (49.0)	4,310 (48.0)	10,755 (48.6)	
**Grade**						*p < * 0.001
I	36,312 (21.1)	34,687 (28.8)	1,203 (5.8)	123 (1.4)	299 (1.4)	
II	71,479 (41.5)	57,862 (48.1)	8,205 (39.8)	1,994 (22.2)	3,418 (15.4)	
III	64,388 (37.4)	27,859 (23.1)	11,235 (54.4)	6,857 (76.4)	18,437 (83.2)	
**Tumor size**						*p < * 0.001
≤2 cm	107,059 (62.2)	82,378 (68.4)	10.644 (51.6)	4,098 (45.7)	9,939 (44.9)	
2–5 cm	55,146 (32.0)	33,023 (27.4)	8,317 (40.3)	3,792 (42.2)	10,014 (45.2)	
>5 cm	9,974 (5.8)	5,007 (4.2)	1,682 (8.1)	1,084 (12.1)	2,201 (9.9)	
**Regional nodes**						*p < * 0.001
Negative	118,127 (68.6)	85,924 (71.4)	12,561 (60.9)	5,078 (56.6)	14,564 (65.7)	
Positive	54,052 (31.4)	34,484 (28.6)	8,081 (39.1)	3,896 (43.4)	7,590 (34.3)	
**Chemotherapy**						*p < * 0.001
NO	95,129 (55.2)	82,944 (68.9)	5,218 (25.3)	1,917 (21.4)	5,050 (22.8)	
YES	77,050 (44.8)	37,464 (31.1)	15,425 (74.7)	7,057 (78.6)	17,104 (77.2)	
**Radiation**						*p < * 0.001
NO	71,202 (41.3)	47,502 (39.5)	9,472 (45.9)	4,442 (49.5)	9,786 (44.2)	
YES	94,620 (55.0)	69,167 (57.4)	10,094 (48.9)	4,076 (45.4)	11,283 (50.9)	
Unknown	6,357 (3.7)	3,739 (3.1)	1,077 (5.2)	456 (5.1)	1,085 (4.9)	

*IQR, interquartile range*.

a *p-value of chi-square test comparing the different subtype groups*.

b *Other: including Asian or Pacific Islander and American Indian/Alaska Native and Unknown*.

## Results

### Patient Demographics and Tumor Characteristics

We identified 172,179 eligible patients from the SEER database according to the aforementioned inclusion criteria. Patient demographics, pathology, and clinical characteristics according to molecular subtype are summarized in Table 1. For the whole cohort, the median follow-up time was 43 months (interquartile range, 26–62 months). Significant differences (*p* < 0.001) were observed in all variables in each of the four different IHC-defined breast cancer subtypes. Elderly patients were a larger component in the HoR(+)/HER2(–) group than in the HoR(+)/HER2(+), HoR(–)/HER2(+), and triple-negative groups (25.2 vs. 16.9, 16.6, and 18.9% of age ≥ 70 years). However, the proportion of patients diagnosed at an earlier age (age < 40 years) was smaller in the HoR(+)/HER2(–) group than in the HoR(+)/HER2(+), HoR(–)/HER2(+), and triple-negative groups (4.7 vs. 9.9, 8.5, and 10.0% were age <40 years, respectively). In terms of tumor characteristics, the HoR(+)/HER2(–) group was associated with lower grade (for grade 1: 28.8 vs. 5.8, 1.4, and 1.4%), smaller tumor sizes (for size ≤ 2 cm: 68.4 vs. 51.6, 45.7, and 44.9%), fewer positive lymph nodes (for positive nodes: 28.6 vs. 39.1, 43.4, and 34.3%), and a lower chemotherapy proportion (31.1 vs. 74.7, 78.6, and 77.2%).

### Survival Analysis of Different Age Groups

**Figure 1:**
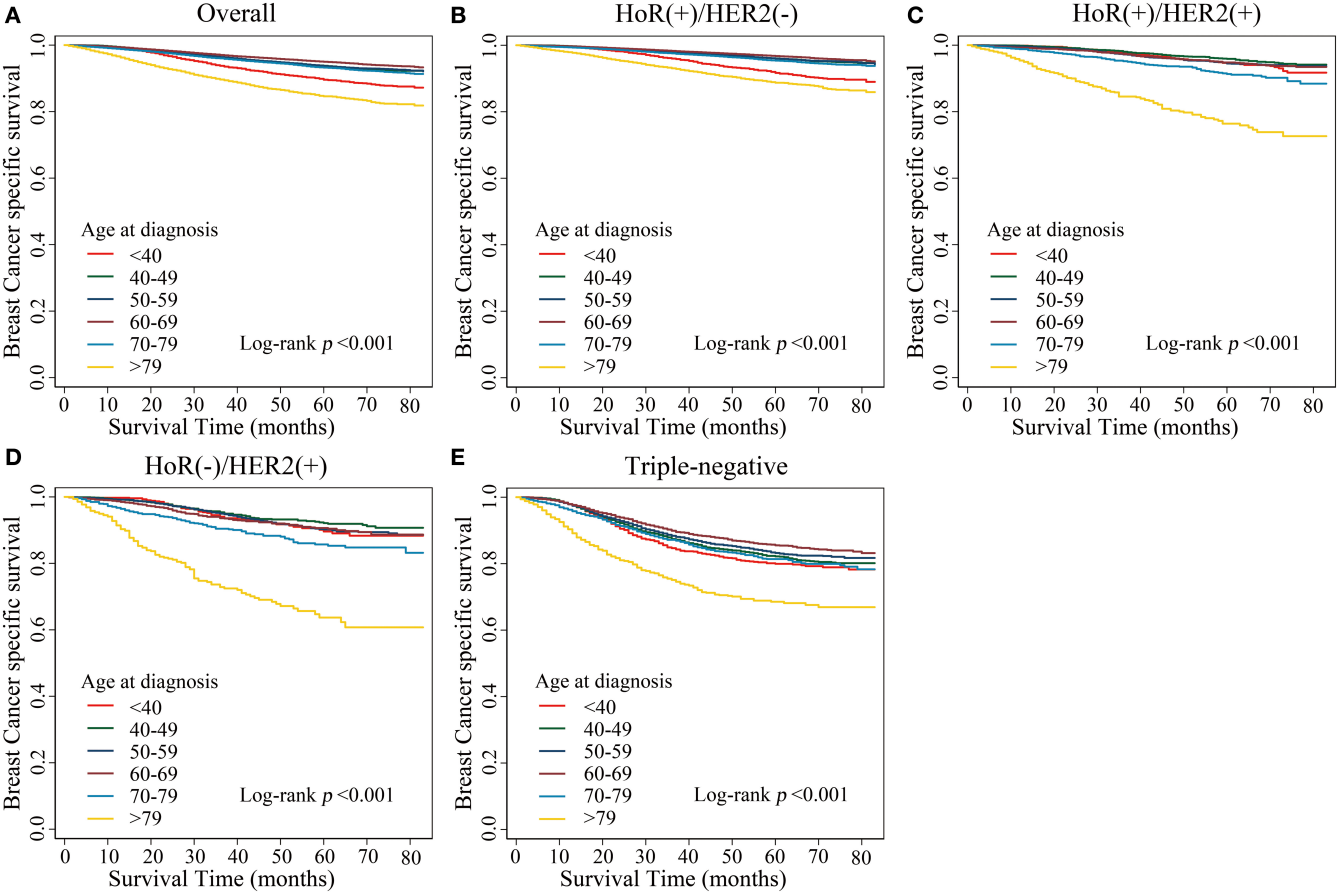
Kaplan–Meier estimates of breast cancer-specific survival in different age groups. **(A)** Overall, **(B)** HoR(+)/HER2(–) subtype, **(C)** HoR(+)/HER2(+) subtype, **(D)** HoR(–)/HER2(+) subtype, and **(E)** Triple-negative subtype.

Kaplan–Meier estimates of breast cancer-specific survival in the different age groups showed that patients aged <40 years and patients aged >79 years presented with the worst survival rates (*p* < 0.001; Figure 1A). We further analyzed breast cancer-specific survival in each subtype and observed that the tendencies of the survival curves differed between patients of different subtypes. In the HoR(+)/HER2(–) subtype, patients aged <40 years showed poor survival rates, similar to that of patients aged >79 years, while the other age groups showed a flatter survival curve (Figure 1B). However, in the HoR(+)/HER2(+), HoR(–)/HER2(+), and triple-negative subtypes, patients aged >79 years showed poor survival rates, while the remaining age groups showed similar survival curves (Figures 1C–E).

Univariate analysis revealed that subtype, age at diagnosis, race, marital status, year of diagnosis, tumor laterality, grade, tumor size, regional lymph node status, and application of chemotherapy and radiotherapy were factors significantly associated with BCSM (Table 2, *p* < 0.01). The group aged 40–49 years presented with the best survival result and was subsequently used as the reference for other age groups in both univariate and multivariate analyses. In the multivariate analysis, the HR of BCSM was 1.13 (95% CI, 1.03–1.23; *p* < 0.01) in the group aged <40 years and was lowest in the group aged 40–49 years. Afterwards, the HR of BCSM began to increase alongside patient age, with the highest HR of 3.52 (95% CI, 3.23–3.83; *p* < 0.01) observed in the eldest age group (aged >79years). The results were consistent with the previous Kaplan–Meier plot analysis.

**Table 2:** Cox proportional hazards regression model analysis of breast cancer-specific mortality.

**Variable**	**Univariate analysis**		**Multivariate analysis**^**a**^	
	**HR (95% CI)**	***p-*value**	**HR (95% CI)**	***p-*value**
**Subtype**				
HoR(+)/HER2(–)	Reference	–	Reference	–
HoR(+)/HER2(+)	1.34 (1.25–1.45)	*p < * 0.01	0.83 (0.77–0.90)	*p < * 0.01
HoR(–)/HER2(+)	2.55 (2.35–2.76)	*p < * 0.01	1.24 (1.13–1.35)	*p < * 0.01
Triple-negative	4.48 (4.27–4.70)	*p < * 0.01	2.42 (2.29–2.55)	*p < * 0.01
**Age at diagnosis**				
<40	1.65 (1.51–1.80)	*p < * 0.01	1.13 (1.03–1.23)	*p < * 0.01
40–49	Reference	–	Reference	–
50–59	0.98 (0.91–1.05)	*p =* 0.51	1.12 (1.05–1.20)	*p < * 0.01
60–69	0.80 (0.75–0.87)	*p < * 0.01	1.19 (1.11–1.28)	*p < * 0.01
70–79	1.10 (1.02–1.19)	*p =* 0.02	1.76 (1.63–1.91)	*p < * 0.01
>79	2.82 (2.61–3.04)	*p < * 0.01	3.52 (3.23–3.83)	*p < * 0.01
**Race**				
White	Reference	–	Reference	–
Black	2.04 (1.93–2.16)	*p < * 0.01	1.34 (1.27–1.42)	*p < * 0.01
Other^b^	0.70 (0.65–0.77)	*p < * 0.01	0.70 (0.64–0.77)	*p < * 0.01
**Marital status**				
Married	Reference	–	Reference	–
Unmarried	1.75 (1.67–1.83)	*p < * 0.01	1.25 (1.20–1.31)	*p < * 0.01
Unknown	1.35 (1.22–1.49)	*p < * 0.01	1.16 (1.05–1.29)	*p < * 0.01
**Year of diagnosis**				
2010	Reference	–	Reference	–
2011	0.97 (0.91–1.03)	*p =* 0.29	0.97 (0.91–1.04)	*p =* 0.42
2012	0.93 (0.87–1.00)	*p =* 0.05	0.96 (0.89–1.02)	*p =* 0.20
2013	0.90 (0.83–0.97)	*p < * 0.01	0.92 (0.86–0.99)	*p =* 0.03
2014	0.96 (0.88–1.04)	*p* = 0.30	1.01 (0.93–1.10)	*p =* 0.30
2015	0.94 (0.84–1.04)	*p* = 0.23	0.98 (0.88–1.09)	*p =* 0.23
**Laterality**				
Left	Reference	–	Reference	–
Right	0.95 (0.91–1.00)	*p =* 0.03	0.97 (0.92–1.01)	*p =* 0.11
**Grade**				
I	Reference	–	Reference	–
II	3.63 (3.21–4.11)	*p < * 0.01	2.31 (2.03–2.62)	*p < * 0.01
III	12.21 (10.84–13.76)	*p < * 0.01	4.58 (4.04–5.20)	*p < * 0.01
**Tumor size**				
≤2 cm	Reference	–	Reference	–
2–5 cm	4.51 (4.28–4.76)	*p < * 0.01	2.31 (2.18–2.44)	*p < * 0.01
>5 cm	12.05 (11.31–12.83)	*p < * 0.01	4.92 (4.59–5.20)	*p < * 0.01
**Regional nodes**				
Negative	Reference	–	Reference	–
Positive	4.24 (4.05–4.44)	*p < * 0.01	2.83 (2.69–2.97)	*p < * 0.01
**Chemotherapy**				
No	Reference	–	Reference	–
Yes	2.14 (2.05–2.24)	*p < * 0.01	0.93 (0.87–0.98)	*p < * 0.01
**Radiation**				
No	Reference	–	Reference	–
Yes	0.56 (0.54–0.59)	*p < * 0.01	0.61 (0.58–0.64)	*p < * 0.01
Unknown	0.94 (0.84–1.05)	*p =* 0.25	0.71 (0.63–0.79)	*p < * 0.01

*HR, hazard ratio; CI, confidential interval*.

a Adjusted by Cox proportional hazards models including all factors, as categorized in

b *Other: including Asian or Pacific Islander and American Indian/Alaska Native and Unknown*.

### Comparison of Survival Between Age and IHC-Defined Subtype

**Figure 2:**
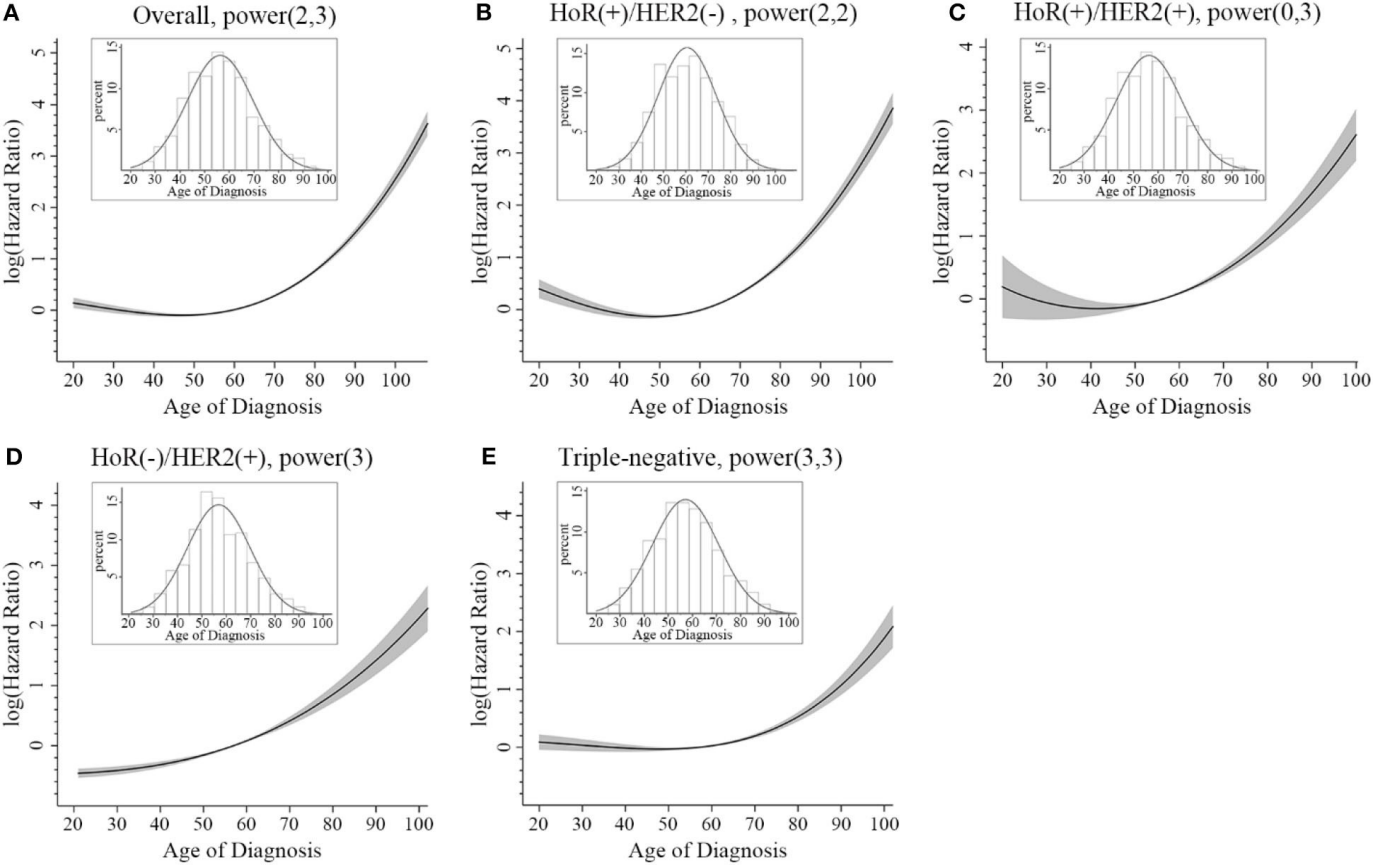
Relative hazard of breast cancer-specific mortality. The thick black line shows the logarithm hazard ratio, and the gray shades show the 95% confidence interval. The superimposed histogram demonstrates that the age distribution is approximately normal. **(A)** Overall, **(B)** HoR(+)/HER2(–) subtype, **(C)** HoR(+)/HER2(+) subtype, **(D)** HoR(–)/HER2(+) subtype, and **(E)** triple-negative subtype.

To investigate whether there was significant interaction between age at diagnosis and IHC-defined breast cancer subtype in predicting BCSM, we utilized an interaction term (i.e., age × subtype). Pairwise comparison between the different combinations of age and subtype showed that in the HoR(+)/HER2(–) subtype, patients aged <40 years had worse BCSM than those aged 40–49 years (HR 1.26; 95% CI, 1.10–1.45; and *p* < 0.01) (Table 3). Similarly, the log hazard ratios for the HoR(+)/HER2(–) patients formed a quadratic relationship between age at diagnosis and BCSM; the lowest risk was approximately around 50 years of age (Figure 2B). Interestingly, the plot in the HoR(+)/HER2(+) subtype seemed to show a U-shaped curve, but the 95% CI was too wide to have a statistical significance (Figure 2C). We observed that in the HoR(–)/HER2(+) subtype, the risk of BCSM was the lowest in patients aged <40 years and increased gradually with age (Figure 2D). However, in the HoR(+)/HER2(+) and HoR(–)/HER2(+) subtypes, patients aged <60 years (including patient aged <40, 40–49, and 50–59 years) had the similar BCSM and exhibited no statistical significant differences (Table 3). HR in the triple-negative subtype was similar between different age groups in patients aged <70 years, but the HR showed significant increase in patients aged over 70 years (Table 3 and Figure 2E).

**Table 3:** Pairwise comparisons between different combinations of age and subtype for breast cancer-specific mortality^a^.

**Age at diagnosis**	**HoR(+)/HER2(–)**		**HoR(+)/HER2(+)**		**HoR(–)/HER2(+)**		**Triple-negative**	
	**HR (95% CI)**	***p* value**	**HR (95% CI)**	***p*-value**	**HR (95% CI)**	***p*-value**	**HR (95% CI)**	***p*-value**
<40	1.26 (1.10–1.45)	*p < * 0.01	1.12 (0.83–1.50)	*p =* 0.45	1.07 (0.75–1.51)	*p* = 0.71	1.03 (0.90–1.18)	*p =* 0.68
40–49	Reference	–	Reference	–	Reference	–	Reference	–
50–59	1.16 (1.04–1.29)	*p < * 0.01	1.34 (1.07–1.68)	*p =* 0.01	1.23 (0.96–1.59)	*p* = 0.10	1.05 (0.94–1.17)	*p =* 0.39
60–69	1.26 (1.12–1.40)	*p < * 0.01	1.48 (1.17–1.88)	*p < * 0.01	1.47 (1.12–1.91)	*p < * 0.01	1.03 (0.92–1.16)	*p =* 0.57
70–79	1.94 (1.73–2.19)	*p < * 0.01	2.27 (1.76–2.92)	*p < * 0.01	2.01 (1.51–2.69)	*p < * 0.01	1.43 (1.25–1.63)	*p < * 0.01
>79	4.19 (3.69–4.75)	*p < * 0.01	4.61 (3.56–5.96)	*p < * 0.01	4.60 (3.41–6.21)	*p < * 0.01	2.21 (1.90–2.57)	*p < * 0.01

*HR, hazard ratio; CI, confidential interval*.

a *The results of different combinations of age (rows) and subtype (columns) are presented in the cross-points of the rows and columns. All results are adjusted by Cox proportional hazards models including race, marital status, year of diagnosis, laterality, grade, tumor size, regional nodes, chemotherapy, and radiation*.

## Discussion

The aim of this study was to explore the relationship between age at diagnosis and BCSM according to IHC-defined breast cancer subtype. Our results suggest that the impact of age upon survival may be more complex than we initially realized. Our study confirmed that the risk of BCSM was lower for patients aged 40–49 years old compared to those aged <40 years, but the risk of BCSM would increase significantly with patients aged ≥50 years.

The association of age at diagnosis with survival in breast cancer has been widely analyzed. Younger age at diagnosis has been reported to be a factor for poor prognosis and is associated with more aggressive disease (17, 18). Previous analyses have found a quadratic relationship between age at diagnosis and BCSM in different subsets (19, 20). Johnson et al. (19) proposed a quadratic relationship between age and the risk of BCSM. Liu et al. (20) proposed a U-shaped relationship between age and the risk of BCSM in the hormone receptor-positive subgroup, which was consistent with our result.

Recent studies have attempted to investigate the influence of age at diagnosis upon prognosis according to different molecular subtypes (12–16). It has been reported that for luminal breast cancer, patients younger than 40 years are more likely to suffer from a significant increase in the risk of BCSM compared with older patients (12). In addition, in the luminal A and luminal B-HER2-negative subtypes, age group younger than 40 years was found to be an independent prognostic factor (13). Liu et al. (14) reported that in the luminal A subtype, patients younger than 40 years had a lower 5-year disease-free survival (DFS) and distant metastasis-free survival (DMFS) compared with the 41–60 years age group, while no significant association of DFS or DMFS with age was found in the other three molecular subtypes. Dai et al. (16) divided patients into the younger group (<40 years) and the older group (≥40 years) and found that the younger group had poorer survival than the older group in the triple negative breast cancer (TNBC) subtype. Despite these conflicting findings, numerous studies support that age at diagnosis is an independent prognostic factor in breast cancer.

In this study, we included the largest number of cases possible from the SEER database in order to determine the effects of age upon survival. However, because the SEER database only provides ER, PgR, and HER2 expression status, we were unable to correctly classify patients into molecular subtypes such as luminal A and luminal B according to current guidelines (4). Therefore, in this study, patients were classified into four IHC-defined breast cancer subtypes, which may cause some disparity between our results and those garnered from studies using molecular subtyping. Our results confirmed that the relationship between age at diagnosis and BCSM showed a quadratic U-shaped pattern only for the HoR(+)/HER2(–) subtype but not for the other IHC-defined breast cancer subtypes.

Different studies have presented several varying ages at diagnosis (such as 45, 50, and 55) as a prognostic factor for the lowest risk (19, 21, 22). Liu et al. (20) took age as a categorical variable and observed that patients aged 40–49 years had the lowest risk of BCSM. In this study, we first treated age at diagnosis as a continuous variable in the fitting model, and we estimated that the minimum risk of BCSM in the HoR(+)/HER2(–) subtype was approximately at the age of 50 years old.

Our results showed that younger HoR(+)/HER2(–) patients aged <40 years had poorer survival rates than patients in the perimenopausal age group. The underlying mechanism is still unclear, but there are several hypotheses to explain this result. For example, premenopausal patients may underestimate the risk of breast cancer at their age, which could lead to a delay in diagnosis and result in later stage disease at initial diagnosis. Another possible explanation is that more aggressive disease may manifest in younger patients, as previous studies have found that patients <40 years of age have a higher histological grade, higher tumor stage, and poorer biological behavior (23). Liu et al. (14) identified 374 differentially expressed genes (DEGs) in the luminal A subtype when divided into two age groups (≤ 40 and >40 years), which were related to breast cancer progression and metastasis, but in the non-luminal A subtypes no age group-specific DEGs were identified. Azim et al. (24) discovered that patients aged ≤ 40 years had a higher expression of RANK-ligand, c-kit, mammary stem cell markers, luminal progenitor markers, and BRCA1 mutation signatures, independent of tumor subtype, grade, and stage. In addition, Morrison et al. (25) reported that in luminal breast cancer subtype patients aged ≤ 40 years, the expression of p53 was significantly higher than in patients aged ≥50 years.

Younger patients with luminal A breast cancer have been shown to have a higher incidence of endocrine resistance (26–28). Even when treated with endocrine therapy, they still may have a poor prognosis due to tamoxifen resistance (26). Young age retains a negative prognostic value particularly in the luminal A subtype (12). A lower incidence and shorter duration of chemotherapy-induced amenorrhea is reported in younger patients and may result in a worse prognosis for hormone receptor-positive breast cancer (29–31). Younger age is also reported to be a predictor of decreased adherence to adjuvant endocrine therapy, associated with increased mortality (32–34). Hershman et al. (32) reported that women <40 years were 40% more likely to be non-adherent to their endocrine treatment than patients aged 50–65 years old (*p* < 0.001). These finding are supported by the results from the SOFT and TEXT trials, which have shown young patients with luminal subtype breast cancer may benefit from a more intensive anti-hormonal (35). Unfortunately, due to the limitations of the SEER database, we were unable to conduct an in-depth analysis of the impact of endocrine therapy upon patient survival. However, considering the fact that patients included in this study were diagnosed from 2010 to 2015, mostly before the results of the SOFT and TEXT trials (36, 37) and before the subsequent renewal of clinical guidelines, it can be expected that most of the premenopausal patients were treated with tamoxifen alone as adjuvant endocrine therapy (38). Therefore, we can estimate that many of these patients were undertreated according to the current standard that recommend the use of ovarian function suppression in many cases (39). This may be a potential explanation as to why HoR(+)/HER2(–) patients aged <40 years presented with poorer outcomes in this study. However, further analysis using a more detailed database containing the specifics of a patient's adjuvant treatment will be needed to support this conclusion.

It has been previously demonstrated that chemotherapy can reduce mortality for many female breast cancer patients, but not for those aged ≥80 years (40). Our results are in concurrence with the previous finding and show that elderly patients had worse disease-specific survival in the overall cohort. Many studies have demonstrated an association between undertreatment and poor survival outcomes (41–43), and it is understandable that with the increase of age, the probability of undertreatment may increase as well. It has been reported in previous studies that older patients are less likely to receive the standard-of-care treatment, including surgical therapy, chemotherapy, adjuvant radiotherapy, and endocrine therapy (44–47). Older patients often have other underlying health issues and may suffer from more serious side effects when receiving standard therapy, which could increase disease-specific mortality in elderly patients (48). In our analysis, the difference in survival among age groups was still significant even after adjustment for radiotherapy and chemotherapy. While the presence of comorbidities may preclude the use of chemotherapy, it is unclear why the application of adjuvant endocrine therapy was suboptimal among eligible elderly women. Unfortunately, we could not control potential confounders such as patient frailty and undertreatment due to the lack of information regarding comorbidities and the incomplete treatment information in the SEER database.

This study was based on the SEER database, which includes cancer incidence and survival information from 18 registries, covering ~27.8% of the U.S. population. However, our study has several limitations. First, as mentioned previously, detailed information regarding patient treatment (for example: whether patient received neoadjuvant or adjuvant chemotherapy, the type of chemotherapy used, the type of endocrine therapy received, whether anti-HER2-targeted therapy was used and whether patient completed radiotherapy) is not recorded in the SEER database, which limits further investigation regarding the impact of therapeutic regimens on clinical outcomes. Second, breast cancer subtypes are roughly defined by ER, PgR, and HER2 status in the SEER database. The lack of data regarding Ki67 expression and other detailed molecular indicators (without which the luminal A and luminal B subtypes could not be properly distinguished according to current standards) only allows us to categorize the patients into four IHC-defined breast cancer subtypes. Third, the median follow-up of this study is only 43 months, and it is possible that our study may have missed late recurrences, which are not uncommon in the luminal and HoR(+) subtype. Finally, because this study utilizes retrospective methodology, sampling bias may have been introduced. Therefore, our results should be confirmed and supplemented by further prospective studies with more information and precise molecular subtypes before clinical application. Despite the limitations mentioned above, our study contributes to the growing evidence that the relationship between age at diagnosis and BCSM varies by tumor subtype.

In conclusion, through analysis of the largest sample size available in the SEER database, the current study showed that the prognostic value of age in determining BCSM varies with IHC-defined breast cancer subtype. Younger age at diagnosis may be particularly prognostic in HoR(+)/HER2(–) breast cancer, but further evidence is needed to analyze the prognostic value of age in premenopausal patients receiving standard adjuvant endocrine therapy. The development of individualized treatment strategies for patients of different ages may be a viable direction for future research, with additional emphasis on intensified treatment for young patients with HoR(+)/HER2(–) breast cancer.

## Data Availability Statement

Publicly available datasets were analyzed in this study. This data can be found here: www.seer.cancer.gov.

## Ethics Statement

The studies involving human participants were reviewed and approved by Lishui Hospital, Zhejiang University School of Medicine. Written informed consent for participation was not required for this study in accordance with the national legislation and the institutional requirements.

## Author Contributions

All authors participated in this research. SCa and SCh: concepts and design. SCa and ZG: data acquisition, analysis, and interpretation. YZ, PL, and YP: material support. SCa and SCh: study supervision. SCa, XL, and WZ: writing, review, and revision of manuscripts. The final manuscript read and approved by all authors.

## Conflict of Interest

The authors declare that the research was conducted in the absence of any commercial or financial relationships that could be construed as a potential conflict of interest.
