# Supplementary material for: The Prognostic Impact of Age at Diagnosis Upon Breast Cancer of Different Immunohistochemical Subtypes: A Surveillance, Epidemiology, and End Results (SEER) Population-Based Analysis
Source: Front Oncol. 2020 Sep 23;10:1729. doi: 10.3389/fonc.2020.01729 (PMC7538776; doi:10.3389/fonc.2020.01729)
Supplement: Supplementary file 1 [file Data_Sheet_1.docx]

Supplementary Material


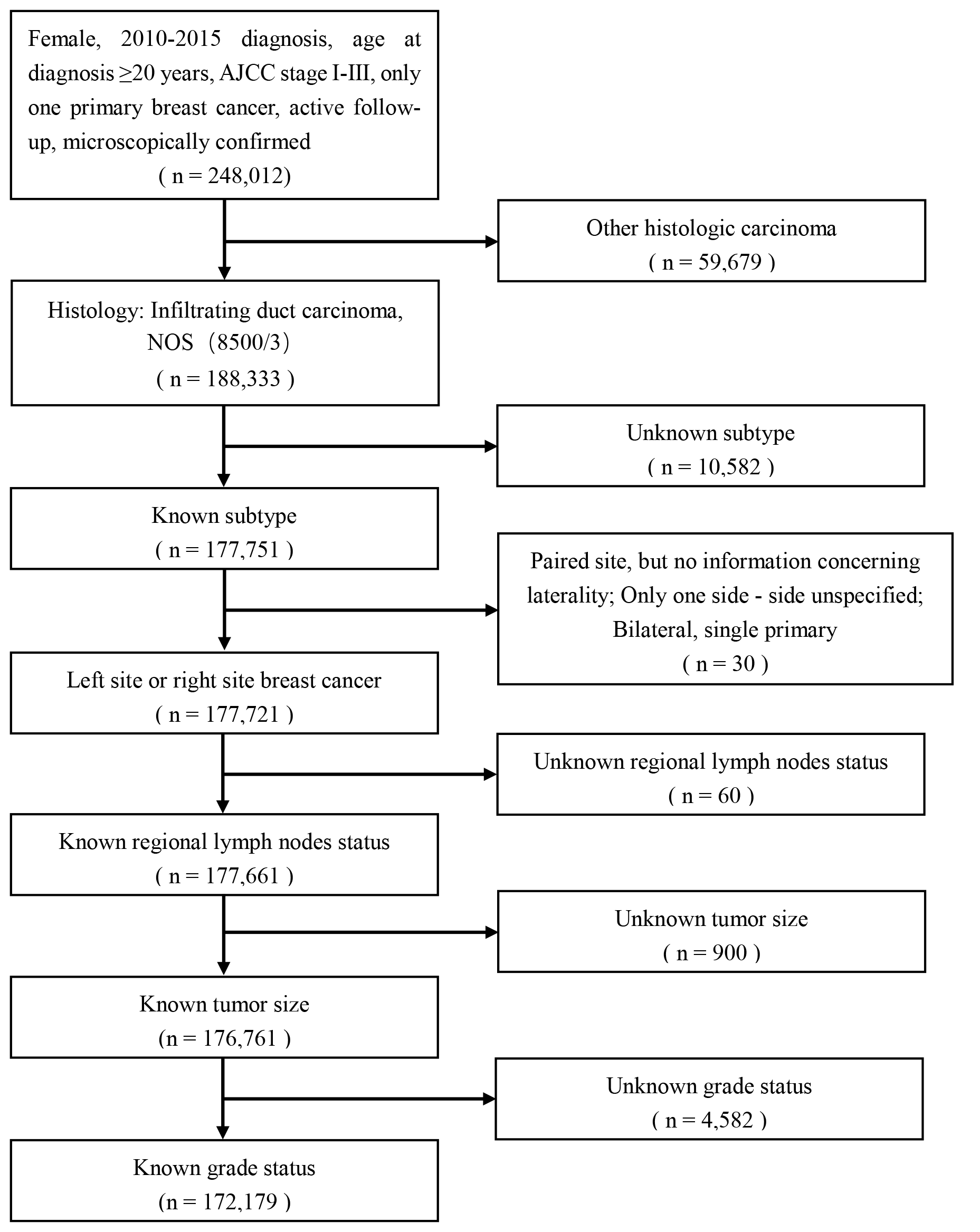


**Supplementary Figure 1** | Flow diagram for selection of the study cohort. A total of 172,179 patients were enrolled in this study.
